# Systematic reviews of animal studies – Report of an international symposium

**DOI:** 10.18849/ve.v8i3.653

**Published:** 2023-08-11

**Authors:** Erik Fausak+, Melissa C. Funaro+, Andrea C. Kespel+, Erin R.B. Eldermire+, Margaret Foster+, Hannah F. Norton+, Kim Mears+, Molly E. Crews+, Marnie Brennan+, Gillian L. Currie+, Megan R. LaFollette+, Annette O'Connor+, Adrian J. Smith+, Kimberley E. Wever+, Suzanne Fricke+

**Keywords:** ANIMAL SYSTEMATIC REVIEW, EVIDENCE SYNTHESIS, EVIDENCE-BASED VETERINARY MEDICINE, LITERATURE SEARCHING, EBVM

## Abstract

Objective: The Symposium on Animal Systematic Reviews held 24 May 2022, sought to bring organisations working on animal literature searching and systematic reviews together into the same virtual space for introductions and discussion.

Background: Groups working on animal research synthesis are often siloed into preclinical, veterinary, and One Health settings. This symposium sought to define commonalities and differences in methodologies, resources, and philosophies and to discuss future needs.

Methods: The 3-hour virtual symposium for veterinarians, researchers, and information specialists began with introductions by panelists from organisations involved in searching the literature for animal studies and conducting systematic reviews. This was followed by a panel discussion and question and answer period.

Results: Panelists identified a need to ensure planning and accurate description of primary animal studies as a precursor to quality systematic reviews. They acknowledged and discussed differences in evidence synthesis expectations and tools based on the type of review, the types of studies available on the topic, and the focus on preclinical, veterinary, or One Health topics.

Conclusion: The need to increase the speed and quality of evidence reviews, and to automate updates, requires investing in the development of both skilled teams and platforms. The symposium provided a chance to identify existing resources, define challenges, and note gaps unique to systematic reviews of animal studies.

Application: This symposium acts as a baseline for ongoing discussions centred on improving the culture and pipeline for evidence syntheses of animal studies that inform decision-making.

## Introduction

Systematic reviews and other evidence syntheses are important tools for summarising and synthesising research from multiple locations into a single, accessible source. In addition to their traditional use in human medicine, these methodologies are increasingly applied in other disciplines, including animal studies. Systematic reviews are particularly important in animal studies due to small study sample sizes, bias associated with individual study populations, and publication silos associated with animal health literature (Manlove et al., 2016). In turn, the creation of systematic reviews helps to identify gaps where further research is needed and drives the use of standardised reporting guidelines so that future studies can more easily be compared with one another.

As more systems become available that incorporate evidence syntheses for veterinary practitioners and animal caregivers, it is crucial that there is a greater understanding of the underlying methodology used. This will enable proper evaluation of these resources for publication and application to policy and practice.

While animal studies share many similarities with human studies, there are distinct factors that need to be considered when conducting and evaluating systematic reviews and meta-analyses of animal studies. The growing interest in systematic reviews supported the creation of a Symposium on Animal Systematic Reviews held on 24 May 2022. Although the symposium focused on systematic reviews, other types of evidence syntheses were discussed. This article summarises discussion at the symposium with a goal of educating veterinary practitioners, animal researchers, systematic review authors, and journal editors and reviewers on current issues in animal evidence synthesis, and provides a curated list of related resources.

## Methods

The Symposium on Animal Systematic Reviews brought together a diverse panel of experts from international organisations and institutions working to advance research, information systems, and syntheses that impact policies and practices affecting the health and welfare of the environment, humans, and animals. This 3-hour virtual symposium included introductions from invited experts, a panel discussion, and an audience question and answer session. The invitation to register for the symposium was promoted through professional organisation listservs, which included veterinarians, animal researchers, and information specialists.

### Objectives

Symposium panelists and participants met with the following objectives:

- To bring together a diverse group of organisations working on systematic reviews of animal studies into the same virtual space for introductions and discussion. 
- To define commonalities among groups working on systematic reviews of animal studies for differing purposes, e.g., drug development, toxicology, veterinary, animal welfare, food safety, sustainability and conservation.
- To identify how systematic review methodology differs across organisations.
- To discuss how information specialists, researchers and journal editors can create a culture that values the use of guidelines in primary animal studies and evidence syntheses.
- To identify and document resources for systematic reviews of animal studies.
- To identify and document common questions and unique problems that arise when searching for animal literature.

### Panel members introduction

Ten panelists were invited to participate in the symposium. The panelists introduced themselves and their respective organisations and illustrated their support for animal literature searching and syntheses.

- Marnie Brennan, Centre for Evidence-based Veterinary Medicine (CEVM), University of Nottingham.
- Jessie Carder, Animal Welfare Information Center (AWIC), USDA National Agricultural Library (NAL).
- Gillian Currie, Collaborative Approach to Meta Analysis and Review of Animal Data from Experimental Studies (CAMARADES), University of Edinburgh.
- Erin Eldermire, Flower-Sprecher Veterinary Library and Evidence Synthesis Service, Cornell University.
- Margaret Foster, Center for Systematic Reviews and Research Syntheses, Texas A&M University School of Medicine.
- Megan LaFollette, North American 3Rs Collaborative (NA3RsC).
- Annette O’Connor, Department of Large Animal Clinical Sciences and Systematic Reviews for Animals and Food (SYREAF), Michigan State University.
- Adrian Smith, National Consensus Platform for the Replacement, Reduction and Refinement of animal experiments (Norecopa). Norway.
- Jesslyn Thay, Centre for Agriculture and Bioscience International (CABI).
- Kim Wever, PreclinicalTrials.eu, International Prospective Register of Systematic Reviews (PROSPERO) guidelines for registering a review of animal studies, and Medical Center Meta-Research Team, Radboud University, The Netherlands.

## Results

### Why systematic reviews of animal studies are needed

Systematic reviews of animal studies are needed to improve clinical decision-making, to assess how research is conducted, and to inform current practice and policy. Systematic reviews confirm, or conversely question, whether conclusions from any one primary study are generalisable and help to explore the heterogeneity between studies in a field. For animal research consumers (veterinary practitioners and policy-makers), this synthesis can serve as a short-cut for keeping up to date on current research; clinicians reading one well-conducted systematic review can gain insights from multiple primary studies and understand their results in context of one another.

In the preclinical research setting, systematic reviews can help advance understanding of human and animal disease and management, and help recognise when research can move from preclinical studies to the next stage of research (Wever et al., 2012). Systematic reviews also highlight areas where laboratory research can be improved or identify systemic barriers to the implementation of interventions (Sena et al., 2014; de Vries et al., 2014; and LaFollette et al., 2017). For example, in preclinical stroke research with animal models, early systematic reviews identified the need to improve research methods and reporting (Banwell et al., 2009). This feedback resulted in advancements made transparent to researchers in subsequent systematic review updates (McCann et al., 2016). 

Systematic reviews and related methodologies of veterinary studies provide critical appraisal of the quality of the available evidence surrounding a clinical question, increase external validity, reduce the risk of bias, and identify gaps in the research. Compared to human-directed research, there is relatively limited research support for agriculture, and even less for veterinary science and companion animal care, so there is an ethical obligation that researchers maximise the quality of primary studies. 

Panelists noted an increasing need for reviews to be rapid and nimble, engaging policy-makers in the process, citing work by Chris Whitty, Chief Medical Officer for England and Chief Medical Adviser to the UK Government that examined the impact of evidence on real-time government decision-making during the pandemic (Whitty & Collet-Fenson, 2021).

### Oversight and funding for systematic reviews of animal studies

Some worry that the growth in systematic review production may involve duplication of efforts and waste of limited time and resources. Panelists agreed on the need to focus future efforts on improving the quality and not the quantity of reviews, and to encourage researchers to ask if a review on that topic has already been done or if a protocol has been registered on their topic. In addition, researchers should demonstrate where their review fits into the canon of previous work on the topic.

Availability of funding, often influenced by institutional or country policies, can determine review topics, as well as whether researchers focus on preclinical, veterinary, global One Health issues, or the United Nations’ Sustainable Development Goals. In The Netherlands, national funding exists for researchers to conduct mentored systematic reviews, and most applicants for this funding are PhD students. An impact study of this Dutch program showed that students’ completion of a systematic review improved the quality of their future primary research, regardless of whether they participated in subsequent reviews (Menon et al., 2021).

While funding structures may exist for preclinical and One Health evidence synthesis projects, panelists observe less funding available for projects focused on veterinary questions, environmental factors, conventional housing, facilities, husbandry, morbidity and mortality, and background genomic factors of species, breeds and strains, except when veterinary studies are tied to industry (Wareham et al., 2017). One model that a large professional organisation can use to remedy this oversight is to adopt a systematic process whereby members or conference attendees are asked to submit questions, information specialists search to see what questions have already been answered, and working groups pursue those questions that remain.

### Types of evidence synthesis

Types of evidence synthesis reviews have proliferated (Grant & Booth, 2009), with a 2019 article categorising 48 separate review types (Sutton et al., 2019). Some commonly used types include scoping reviews, systematic reviews, rapid reviews, umbrella reviews, and meta-analyses. Panelists shared their perspectives on the continuum of evidence synthesis types with which they have engaged, from systematic reviews to the less rigorous critically appraised topics (Brennan et al., 2020).

Preliminary stakeholder discussions about conducting higher levels of evidence synthesis like systematic reviews and meta-analyses can be challenging depending on the discipline, type of question, and study type availability. For instance, while randomised controlled trials are considered the most reliable type of evidence, this study design is not feasible or ethical in all fields of research. Researchers may lack or underestimate the resources needed to conduct a systematic review. While selling the idea of evidence synthesis is easy, learning the process and defining the scope may be difficult for researchers that are new to the methodology.

The large variety of types of evidence synthesis can create a hurdle for readers, as well as those conducting and reviewing evidence syntheses. Several excellent articles explaining the meaning and purpose of various types of reviews have been published for veterinarians (O’Connor & Sargeant, 2015; and Sargeant & O’Connor, 2014). However, some of the burden falls squarely on authors and journals to better identify review types. Even Centre for Evidence-based Veterinary Medicine (CEVM) expert researchers experience difficulty determining what publications qualify as a systematic review for inclusion in VetSRev (Centre for Evidence-based Veterinary Medicine, 2021), a database that indexes veterinary systematic reviews identified from CAB Abstracts and MEDLINE.

### Reporting guidelines: ensuring quality of primary studies

High-quality evidence synthesis starts with assuring the quality of the primary animal studies that will later be included in syntheses. To improve animal welfare and make use of the limited funding focused on outcomes for animals, planning of the research study design is vital. The PREPARE (Planning Research and Experimental Procedures on Animals: Recommendations for Excellence) guidelines include a two-page checklist available in over 30 languages for planning animal studies (Smith, 2018). Planning is aided by a culture that encourages the pre-registration of animal primary study protocols (Baker, 2019; and Moore et al., 2021). Preclinicaltrials.eu (van der Naald et al., 2022) and animalstudyregistry.org (Bert et al., 2019) are dedicated registries for primary animal studies, or authors may use general study registries identified in S1 Table 1. For veterinary studies, the American Veterinary Medical Association maintains the Animal Health Studies Database (Murphey, 2019), modeled on clinicaltrials.gov (US National Library of Medicine, 2000), for recruiting participants.

The use of reporting guidelines for primary studies, that define the information that is necessary to include based on study design, is crucial for comparing one animal study to another in systematic reviews. Well-known preclinical reporting guidelines for animal research are the ARRIVE 2.0 (Animal Research: Reporting of In Vivo Experiments) guidelines (Percie du Sert et al., 2020). Other reporting guidelines can be found through the Enhancing the QUAlity and Transparency Of health Research (EQUATOR) Network (UK EQUATOR Centre, 2019), Menagerie of Reporting Guidelines Involving Animals (MERIDIAN) Network (MERIDIAN Network, 2023), and Norecopa (Norecopa, n.d.), which collect and provide links to a broader number of reporting guidelines applicable to other types of animal research. Authors of primary animal studies should be aware that the design impacts whether a study is included in larger evidence synthesis projects. For instance, a study may not be included in a systematic review if it does not meet the required inclusion / exclusion standards or risk of bias assessment criteria.

While ARRIVE 2.0 and other guidelines include recommendations related to abstracts, the panel highlighted abstracts as a major area where further improvement is needed. Abstracts of primary animal studies are often vague in both technical and plain language. Improvements in abstracts and reporting standards for conference proceedings would also help with efficiency in identifying studies appropriate for inclusion in evidence synthesis projects, since veterinary research is often reported in conference proceedings.

### Preparation for conducting evidence synthesis

- Evidence synthesis training 

An array of toolkits and templates are available to introduce the preclinical systematic review process to first-time evidence synthesis authors. Panelists recommend both the CAMARADES Preclinical Systematic Review Wiki (CAMARADES Berlin, n.d.) and the Systematic Review Center for Laboratory animal Experimentation (SYRCLE) course (SYRCLE, 2023).

RCVS Knowledge Evidence-based Veterinary Medicine (EBVM) Learning (RCVS Knowledge, n.d.[a]) and *Veterinary Evidence* ‘How to Write a Knowledge Summary’ (*Veterinary Evidence*, n.d.[a]) are good starting points for veterinarians. The veterinary systematic review process has been further described in a special issue of the journal *Zoonoses and Public Health* (Torrence (ed), 2014).

Once familiar with systematic review methodologies, researchers exploring more advanced automated aspects of reviews may start with the Evidence Synthesis Hackathon and the accompanying Evidence Synthesis & Meta-Analysis in R Conference (Westgate & Haddaway, 2017; and Haddaway et al., 2022).

- Selecting a review type

Choosing the appropriate review type is a pivotal first step for researchers, as it helps to determine the scope and focus of the review, as well as the methodology and analysis that will be used. Cornell University Library’s Evidence Synthesis Service Review Methodology Tree (Cornell University Library, n.d.), for deciding on a review type, can be a helpful tool to aid researchers in their decision. The flowchart helps researchers understand the basics of each review type and why one might be chosen over another.

- Search development

To assure transparency in the study selection process, PRISMA 2020 guidelines (Page et al., 2021) and the PRISMA-S extension (Rethlefsen et al., 2021) call for authors to identify all information sources searched or consulted, and to provide full search strategies for each. Since animal research is published in a variety of disciplines and half of veterinary research is published outside of core veterinary journals (Grindlay et al., 2012; and Page et al., 2014), this requires searches across multiple databases and platforms with differing features and filters. Also, articles found through citation searching may not be in journals indexed in larger databases, raising concerns that the journal might be predatory.

Panelists voiced how difficult it is to create concise database search strategies, specifically for studies about animal health, versus animal models of human disease. Acronyms or terms that have context outside of animal studies can also be problematic in searches, returning irrelevant results.

- Registration of animal evidence syntheses

Prospective registration of evidence syntheses contributes to methodological transparency and minimises duplication of effort. Panelists addressed what elements of a review should be pre-registered and where they should be registered. Protocol registration requirements developed for human study platforms often do not accommodate animal studies. For instance, clinical trials with small enrollments of six animals, or without controls, would not fulfill Cochrane inclusion requirements. Two tools specifically designed to register protocols for systematic reviews of animal studies include the guidelines developed for preclinical systematic review registration (UK CRD, n.d.) in the human-centric International Prospective Register of Systematic Reviews (PROSPERO) (Booth et al., 2012), and Systematic Reviews for Animals and Food (SYREAF) Protocols (SYREAF, 2023).

PROSPERO registers systematic reviews, rapid reviews or umbrella reviews of preclinical animal research studies that are of ‘direct relevance to human health’. PROSPERO does not register scoping reviews. Developed with input from researchers, PROSPERO’s preclinical form includes more mandatory fields than the clinical form. The PROSPERO team does not perform formal peer-review of protocols, but checks for transparency of methods and completeness of information.

Systematic review protocols of animal studies without a direct relevance to human health, such as those in veterinary or agricultural sciences, can be registered with SYREAF Protocols or Open Science Foundation (OSF) Registries (Center for Open Science, n.d.). The SYREAF team currently considers strict guidelines as a barrier to veterinary researchers submitting protocols, therefore few protocol elements are mandatory.

Some additional conversation among attendees highlighted researchers' hesitancy to pre-register protocols due to concerns related to intellectual property. Panel members felt that researchers fearful of protocol registration were in the minority. They asserted that pre-registration serves as a means to 'claim' a particular topic and that many journals and reviewers expect and reward this practice. Pre-registration of animal systematic review protocols also encourages the pre-registration of primary animal study protocols. 

- Study types

Types of studies to include in animal evidence synthesis projects depends both on the available research in a particular discipline and the type of question being asked in the review. There may be an abundance of clinical trials in some disciplines, and in others the body of evidence may be much more qualitative or rely heavily on observational studies. For instance, preclinical research questions, focused on mechanisms of disease, therapeutic targets, and safety, translate best to randomised and controlled experimental studies, while research questions in clinical veterinary settings concerned with the aetiology, prevention, or prognosis of a disease translate best to observational studies (*Veterinary Evidence* ‘Guidance for writing the clinical bottom line,’ n.d.[b]). Broader One Health synthesis projects may include multiple questions with multiple variables, often incorporating mixed-method studies that address the role of human attitudes and behaviour. What might be evidence in one discipline does not always apply to all disciplines.

- Tools and resources

Panelists noted that there are many tools for conducting systematic reviews. However, tools are only a means to an end and selecting an effective tool will depend on funding, familiarity, and unique needs of the review topic and / or field. Preclinical animal researchers, in particular, value traditional systematic review screening tools such as Covidence (Covidence, n.d.), Rayyan (Rayyan, n.d.), and DistillerSR (DistillerSR, n.d.). However, some screening tools designed for human studies cannot handle common animal study protocols. For example, many popular screening tools such as Covidence, do not support veterinary studies that include multiple primary studies in one publication. To facilitate the creation of preclinical *in vivo* systematic reviews, Systematic Review Facility (SyRF) was created to decrease the time needed for reviews and the need to use multiple software programs (Bahor et al., 2021).

A question was raised about whether the differences between animal and human systematic reviews are distinct enough to justify the need for different tools and resources. Panel member opinions varied about the degree to which different tools are required across preclinical, veterinary, and human studies, however all panelists agreed that, at this point in time, human-centric tools do not adequately support evidence synthesis of animal studies. This may be an important consideration as the number of One Health studies continues to grow.

### Living systematic reviews

As the amount of evidence grows and as global questions become more complex and incorporate multiple variables, more organisations are pursuing continuously updated living systematic review platforms, which have an *a priori* plan to incorporate new evidence over time. 

CAMARADES and SYREAF have both explored living systematic reviews made available via R Shiny web applications. CAMARADES has implemented living systematic reviews on transgenic mice models of Alzheimer’s Disease and Covid-19 research (Hair et al., 2021b), and SYREAF has a living systematic review looking at the impact that multiple effects of animal production have on the health of surrounding communities (Fonseca & O’Connor, 2021 and SYREAF, 2023).

CAMARADES developed Systematic Online Living Evidence Summaries (SOLES) for different research areas by implementing automation and crowd sourcing (Hair et al., 2021b). Automation adds the ability to analyse data at a level that surpasses that of human screeners (Bannach-Brown et al., 2019). Living systematic reviews are greatly facilitated when workflows and platforms are created that minimise a need to transfer records from one tool to another.

### Educating the next generation of practitioners on evidence synthesis

Panelists acknowledged a distinction between authors and consumers of systematic reviews and approach each audience differently. Practitioners and students may not conduct research or become authors of reviews, but they need to know about different review types and how to apply their findings to practice. Because of this, it is important to integrate instruction on using systematic reviews into the curricula of healthcare professions. The RCVS Knowledge EBVM Learning Course teaches veterinary practitioners how to apply evidence to answer clinical questions, which is important foundational knowledge when engaging in the review process. Students and first-time authors can engage first with ‘mini reviews’ called critically appraised topics (CATs), a type of rapid review (Brennan et al., 2020). Clinical examples include Knowledge Summaries published in *Veterinary Evidence *(RCVS Knowledge, n.d.[b]) and BestBETs for Vets (University of Nottingham, 2023) made available online through the CEVM. These are inexpensive, time efficient and simple ways of doing reviews that are useful for both learners and clinicians. 

Depending on their availability, students across a variety of disciplines and levels of education can contribute significantly to a larger synthesis project. Involving a few undergraduates in phases of a synthesis review (as opposed to the entire process) can provide substantive experience and perhaps material for an honours degree thesis. Graduate students, or professional students enrolled in concurrent degree programmes such as public health, will dive deeper into scholarly writing as part of their curriculum. By forming a cohort, these students can peer-review each other’s work to strengthen the learning process and to learn to apply reviews across different questions and topics. This then requires fewer one-on-one consultations by mentors or advisors. Building capacity for conducting systematic reviews among the next generation is vital to the continued evolution and improvement of review methods.

### Artificial intelligence and the future of systematic reviews

Evidence synthesis projects rely increasingly upon machine learning and artificial intelligence (AI). It may become harder to perform evidence synthesis without automation tools that create and translate searches, citation search, deduplicate citations, screen both title / abstracts and full-text, extract data, assess risk of bias, and manage workflows. AI tools are needed to decrease the time required to complete a systematic review. Rapid completion of reviews is critical for policy change, as policy-makers will not often tolerate long timelines. In the health and social sciences, detailed manuals and handbooks, such as the Cochrane Handbook for Systematic Reviews of Interventions and the Campbell Collaboration’s guidelines, contain rigorous methodological guidance on the process of conducting evidence syntheses. However, researchers using AI in evidence syntheses are likely to come from diverse research fields where detailed guidance may not exist. With this in mind, a network of organisations, the International Collaboration for the Automation of Systematic Reviews (ICASR), works to create open tools for automating evidence synthesis (Beller et al., 2018).

The panelists outlined some of the synthesis review tasks where AI tools have been deployed for evidence synthesis projects involving animal studies. Not all available tools were discussed in the symposium, and further recommendations for AI use in evidence synthesis are defined elsewhere (Bersani et al., 2022). 

Tools include those for: 

- Search strategy development

Text mining and keyword co-occurrence networks (KCNs) using R package litsearchr can help automate identification of keywords for search strategy development, particularly in fields where standard controlled terminologies do not exist (Grames et al., 2019). The SR-Accelerator Polyglot Search Translator can help translate search strings into the syntax of other databases (Clark et al., 2020a; and Clark et al., 2020b).

The use and accessibility of generative AI search tools has exploded since this symposium was held in 2022, and information specialist authors caution that with many of these new tools the search is not replicable and relies on simple searches that can perpetuate existing silos within the literature. They do not adequately capture the semantic search combinations of controlled vocabulary and keywords needed to comprehensively search literature across the various disciplines, databases, and indexing and metadata conventions associated with animal studies. Therefore, these tools should be seen as accessories to collaborative search strategy development by human teams familiar with both the topic and database querying.

- Citation searching 

Several AI tools for backwards and forwards citation searching exist, including DistillerSR, Citation Chaser and SR Accelerator SpiderCite (DistillerSR, n.d.; Haddaway et al., 2021; and Institute for Evidence-Based Healthcare, n.d.). 

- Deduplication 

The CAMARADES team uses Automated Systematic Search Deduplicator (ASySD), a tool for deduplication that they developed themselves (Hair et al., 2021a). Other software such as Covidence, Rayyan, and DistillerSR also have a built-in deduplication function.

- Screening 

Effective tools that facilitate screening of articles for inclusion or exclusion at the title and abstract level have been developed for many platforms including SyRF, DistillerSR, SR-Accelerator, Covidence and others. Systematic review screening standards dictate that more than one reviewer evaluates citations in order to eliminate bias. However, screening can be time consuming. Organisations have approached the time barrier that screening presents in a variety of ways. CAMARADES has crowdsourced and trained a large pool of reviewers, while others have explored the potential of AI to replace some human reviewers.

Confirming the difficulty in identifying study design, the CAMARADES and SYREAF teams have both tried to automate the detection of study design in biomedical research and were not successful (O’Connor et al., 2018). However, the CAMARADES team has developed an algorithm hosted by the Evidence for Policy and Practice Information and Coordinating Centre (EPPI-Centre) at University College London to classify studies based on title and abstract screening as ‘primary’ or ‘other’ (Hair et al., 2021b).

- Extraction

The SYREAF team and other panelists highlighted text extraction from PDFs as one of the most difficult automation steps. SyRF has worked on a graphical data extraction tool to aid in meta-analysis of preclinical studies (Bahor et al., 2021). 

- Risk of bias assessment

Automated tools that assess the actual risk of bias are difficult to develop, since performing a risk assessment is much more complex than extracting experimental variables. The reporting of measures to reduce bias, such as the term ‘blinded experimenter’ or ‘random allocation’ can be more readily assessed with automated techniques. The SYRCLE Risk of Bias (RoB) tool was developed to assess risk of bias for preclinical trials (Hooijmans et al., 2014). Evidence on the efficacy of AI tools in evidence synthesis for assessing quality of publications is lacking.

## Discussion

Systematic reviews are useful tools for synthesising the results of studies, identifying research gaps, informing future research, and reducing research waste. As such, they are beneficial for health practitioners, researchers, and policy-makers. Systematic reviews based on studies of animals are increasingly being recognised as a critical part of knowledge translation frameworks by preclinical, veterinary and One Health groups that have not formally come together to discuss evidence synthesis.

What constitutes a high-quality systematic review is an evolving metric. Those working on synthesis of animal studies face common barriers of search strategy terminology and studies with small sample size, though the symposium uncovered distinct differences across disciplines in supporting infrastructures, required steps of the review, and included study designs. While much has already been done to define appropriate methodologies and create freely available tools, there are areas where development is still needed in order to further animal-focused evidence syntheses (e.g., inclusion of observational studies in reviews, AI). Automation of steps has the potential to make conducting systematic reviews more accessible to smaller research teams. 

When looking at the breadth of topics under the 'evidence synthesis of animal studies' umbrella, recognising the value of having teams with individual skill sets involved in reviews (e.g., information specialists, subject expert researchers and clinicians) is key. Capturing discussions like this between various organisations, teams and individuals working to synthesise heterogenous studies contributes to a culture aiming to strengthen both evidence synthesis and primary research design and reporting.

The Symposium on Animal Systematic Reviews provided a unique opportunity for preclinical and clinical researchers, and information specialists, to collectively assess the state of systematic reviews focused on animals. While the underlying symposium objectives served to guide discussion, the time limited format did not allow for complete stakeholder representation or consensus building, particularly in the area of One Health. This was instead a forum for varied stakeholders, both panelists and attendees, to identify common challenges and opportunities unique to evidence synthesis of animal studies, including the use of existing resources or the need to develop new resources. Resources discussed in the symposium are compiled and shared in S1 Table 1.

Conversations and discussions should continue to further address some of the issues and barriers identified, including abstracts, indexing and reporting guidelines for primary studies, pre-registration, roles for AI, and types of studies to include, in order to improve the speed and quality of evidence synthesis of animal studies.

## Supplementary materials

Supplementary Material S1 – Table 1: List of selected resources for evidence synthesis of animal studies discussed in the symposium.

### Acknowledgements

The authors credit the Medical Library Association (MLA), and members of the Animal and Veterinary Information Specialist Caucus (AVIS) of MLA, the United States Agricultural Information Network (USAIN), the European Veterinary Libraries Group (EVLG) of the European Association for Health Information and Libraries (EAHIL), the Evidence-Based Veterinary Medical Association (EBVMA), and multiple research organisations and institutions for promoting the event. The authors would like to thank Elizabeth Tobey and Jessie Carder with USDA AWIC, and Jesslyn Thay with CABI, for their help with the symposium.

### Author contributions

**Erik Fausak:** Conceptualisation, Methodology, Writing – Original draft preparation, Writing – Review and Editing. **Melissa C. Funaro:** Conceptualisation, Methodology, Writing – Original draft preparation, Writing – Review and Editing. **Andrea C. Kepsel:** Conceptualisation, Methodology, Writing – Original draft preparation, Writing – Review and Editing.  **Erin R.B. Eldermire:** Conceptualisation, Methodology, Investigation, Writing – Review and Editing. **Margaret Foster:** Conceptualisation, Methodology, Investigation, Writing – Review and Editing. **Hannah F. Norton:** Conceptualisation, Methodology, Writing – Original draft preparation, Writing – Review and Editing. **Kim Mears:** Conceptualisation, Methodology, Writing – Review and Editing. **Molly E. Crews:** Conceptualisation, Methodology, Writing – Review and Editing. **Marnie Brennan:** Investigation, Writing – Review and Editing. **Gillian L. Currie:** Investigation, Writing – Review and Editing. **Megan R. LaFollette:** Investigation, Writing – Review and Editing. **Annette O’Connor:** Investigation, Writing – Review and Editing. **Adrian J. Smith:** Investigation, Writing – Review and Editing. **Kimberley E. Wever:** Investigation, Writing – Review and Editing. **Suzanne Fricke:** Conceptualisation, Methodology, Writing – Original draft preparation, Writing – Review and Editing, Funding Acquisition, Supervision, Project administration.

### ORCID

Erik Fausak: https://orcid.org/0000-0002-0510-0153

Melissa C. Funaro: https://orcid.org/0000-0001-6846-4846

Andrea C. Kepsel: https://orcid.org/0000-0002-9651-6700

Erin R.B. Eldermire: https://orcid.org/0000-0001-5846-4041

Margaret Foster: https://orcid.org/0000-0002-4453-7788

Hannah F. Norton: https://orcid.org/0000-0001-8062-2763

Kim Mears: https://orcid.org/0000-0001-6461-9054

Molly E. Crews: https://orcid.org/0000-0002-4960-5591

Marnie Brennan: https://orcid.org/0000-0002-4893-6583

Gillian L. Currie: https://orcid.org/0000-0003-3052-2929

Megan R. LaFollette: https://orcid.org/0000-0003-0643-2210

Annette O’Connor: https://orcid.org/0000-0003-0604-7822

Adrian J. Smith: https://orcid.org/0000-0002-8375-0805

Kimberley E. Wever: https://orcid.org/0000-0003-3635-3660

Suzanne Fricke: https://orcid.org/0000-0002-4412-9717

### Funding

The Symposium on Animal Systematic Reviews received funding from the Medical Library Association.

### Conflict of Interest

Participants were approached to be on the discussion panel based on their personal or organisational involvement with evidence synthesis of animal studies. As part of the discussion, participants were encouraged to mention resources or initiatives familiar to them, which included those originating from themselves or their organisations.

## Custom Section

Custom section here

## Disclaimer

Any opinions expressed in articles and other publication types published in Veterinary Evidence are the author's own and do not necessarily reflect the view of the RCVS Knowledge. Veterinary Evidence is a resource to help inform, and the content herein should not override the responsibility of the practitioner. Practitioners should also consider factors such as individual clinical expertise and judgement along with patient’s circumstances and owners’ values. Authors are responsible for the accuracy of the content. While the Editor and Publisher believe that all content herein are in accord with current recommendations and practice at the time of publication, they accept no legal responsibility for any errors or omissions, and make no warranty, express or implied, with respect to material contained within. For further information please refer to our Terms of Use.
